# Pleiotropic effects of NOACs with focus on edoxaban: scientific findings and potential clinical implications

**DOI:** 10.1007/s00399-023-00944-5

**Published:** 2023-05-04

**Authors:** Andreas Goette, Martin Mollenhauer, Volker Rudolph, Mathias Lamparter, Martin Meier, Michael Böhm

**Affiliations:** 1 Medizinische Klinik II: Kardiologie und Intensivmedizin, St. Vincenz-Krankenhaus Paderborn, Am Busdorf 2, 33098 Paderborn, Germany; 2 Department of Cardiology, Faculty of Medicine and University Hospital Cologne, University of Cologne, Cologne, Germany; 3 Center for Molecular Medicine Cologne (CMMC), University of Cologne, Cologne, Germany; 4 Herz- und Diabeteszentrum Nordrhein-Westfalen, Universitätsklinik der Ruhr-Universität Bochum, Bochum, Germany; 5 Daiichi Sankyo Europe GmbH, Munich, Germany; 6 Daiichi Sankyo Deutschland GmbH, Munich, Germany; 7 Innere Medizin III—Kardiologie, Angiologie und internistische Intensivmedizin, Universitätsklinikum des Saarlandes und Medizinische Fakultät der Universität des Saarlandes, Homburg, Germany

**Keywords:** Anti-inflammatory effects, NOACs, PAR, Thrombin, Factor Xa, Anti-entzündliche Effekte, NOAK, PAR, Thrombin, Faktor Xa

## Abstract

Non-vitamin K antagonist oral anticoagulants (NOACs) are well-established as inhibitors of factor Xa (FXa) and thrombin in the treatment and prevention of thrombosis. However, there is growing evidence that beneficial outcomes might be based on additional pleiotropic effects beyond anticoagulation. FXa and thrombin are also known to activate protease-activated receptors (PARs), which can mediate pro-inflammatory and pro-fibrotic effects. Since PAR‑1 and PAR‑2 play an important role in the development of atherosclerosis, the inhibition of this pathway represents an interesting potential target for preventing the progression of atherosclerosis and fibrosis. This review focuses on potential pleiotropic effects of FXa inhibition with edoxaban seen in a variety of studies in different in vitro and in vivo test systems. As common findings from these experiments, edoxaban was able to attenuate FXa- and thrombin-induced pro-inflammatory and pro-fibrotic effects and decrease pro-inflammatory cytokine expression. In some, but not all experiments edoxaban was also shown to decrease the levels of PAR‑1 and PAR‑2 expression. Further studies are required to clarify the clinical implications of the pleiotropic effects mediated by NOACs.

## Abstract (de)

Nicht-Vitamin-K-antagonistische orale Antikoagulanzien (NOAK) sind als Inhibitoren von Faktor Xa (FXa) und Thrombin in der Behandlung und Prävention von Thrombosen etabliert. Es gibt jedoch zunehmend Hinweise darauf, dass die positiven Ergebnisse auf zusätzlichen pleiotropen Effekten beruhen, die über die Antikoagulation hinausgehen. Faktor Xa und Thrombin können proteaseaktivierte Rezeptoren (PAR) aktivieren, welche proinflammatorische und profibrotische Wirkungen vermitteln. Da PAR‑1 und PAR‑2 eine wichtige Rolle bei der Entstehung von Atherosklerose spielen, stellt die Hemmung dieses Signalwegs ein interessantes potenzielles Ziel dar, um das Fortschreiten von Atherosklerose und Fibrose zu inhibieren. Diese Übersichtsarbeit konzentriert sich auf die potenziellen pleiotropen Effekte der FXa-Hemmung durch Edoxaban, die in einer Vielzahl von Studien in verschiedenen In-vitro- und In-vivo-Testsystemen beobachtet wurden. Übereinstimmende Ergebnisse dieser Studien zeigten, dass Edoxaban die durch FXa und Thrombin induzierten proinflammatorischen und profibrotischen Effekte abschwächen und die Expression proinflammatorischer Zytokine verringern konnte. In einigen, aber nicht allen Experimenten konnte Edoxaban auch die Expression von PAR‑1 und PAR‑2 reduzieren. Weitere Studien sind erforderlich, um die klinischen Auswirkungen der von NOAKs vermittelten pleiotropen Effekte zu untersuchen.

## Introduction

**Fig. 1:**
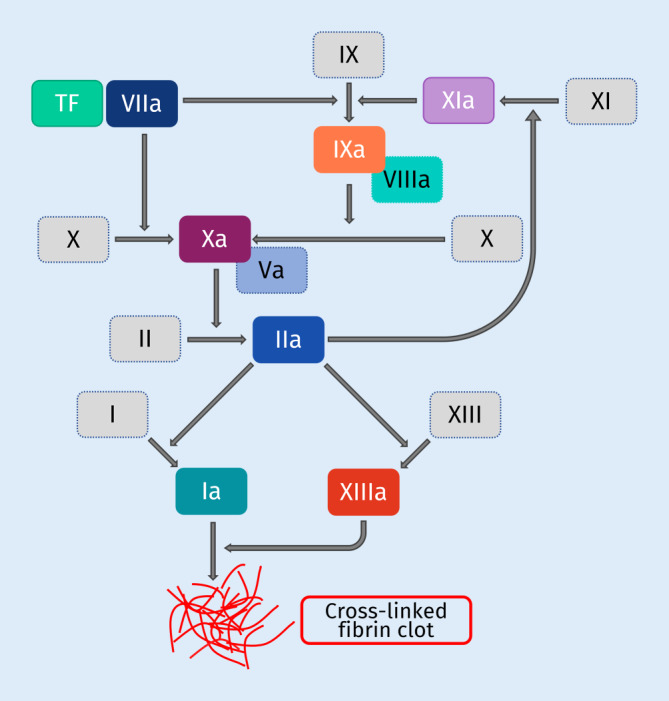
Role of thrombin and factor Xa in haemostasis. Activation of factor Xa and thrombin (*IIa*) as key players in haemostasis ultimately results in the formation of cross-linked fibrin clots. Main physiological activator of the coagulation cascade is tissue factor. *TF* tissue factor

Factor Xa (FXa) inhibitors are well-established in the treatment and prevention of stroke and systemic embolism in patients with atrial fibrillation (AF) and are recommended as first-line anticoagulants for the prophylaxis of thromboembolic events in current guidelines [10, 14, 20, 39, 40]. AF induces hypercoagulability, formation of thrombi within the left atrial appendage, and prothrombotic endothelial changes, leading to significantly increased morbidity and mortality [4, 13, 15]. The activated factor X is a key player in haemostasis, is associated with activated factor V and phospholipids, and activates factor II (prothrombin) to thrombin (FIIa) (Fig. 1; [7]). There is growing evidence for the non-haemostatic effects of thrombin and FXa which are mediated via activation of G‑protein coupled protease-activated receptors (PARs) [6, 28]. Four known isoforms of protease-activated receptors, PAR-1–4, are expressed in various cell types at different expression levels. Thrombin activates PAR‑1 (as well as PAR‑3 and PAR-4), which is mainly expressed in smooth muscle cells, cardiomyocytes, fibroblasts and endothelial cells, but also in platelets and leukocytes [2, 9, 36]. FXa is an activator of PAR‑1 and PAR‑2 and was detected in endothelial cells, smooth muscle cells and myocytes, while its expression in fibroblasts is unclear [2, 36]. Activation of PAR receptors exerts pro-inflammatory and pro-fibrotic effects in several cell types mediating atherosclerosis, atrial remodeling, cardiac hypertrophy and chronic inflammatory pulmonary disorders [6, 33, 36].

**Fig. 2:**
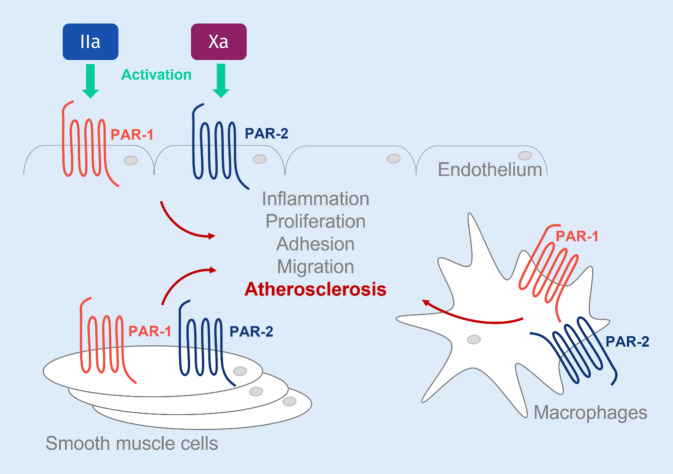
The role of protease-activated receptors in atherosclerosis. Factor Xa and thrombin induce activation of the protease-activated receptors *PAR‑1* and *PAR‑2* on endothelial cells, vascular smooth muscle cells, and macrophages. Increased inflammation, proliferation, cell adhesion and migration are the consequence, resulting in the development and progression of atherosclerosis

PAR‑1 and PAR‑2 are discussed as key receptors involved in atherosclerosis [37]. PARs are activated via serine proteases such as thrombin and FXa via cleavage of the receptor’s N‑terminus, with the N‑terminal cleavage product becoming a ligand of the PAR itself [9, 19]. The cleavage position depends on the protease and is crucial for the signaling pathway. At low concentrations, thrombin activates protein C, which binds to the endothelial protein C receptor (EPCR) and thereby activates co-localized PAR‑1. This mediates protective and anti-inflammatory effects on the endothelium. At high thrombin concentrations, PAR‑1 is activated directly by thrombin via N‑terminal cleavage and mediates pro-inflammatory effects (Fig. 2). FXa can also bind to EPCR, activate PAR‑1 and PAR‑2 via N‑terminal cleavage, and thereby initiate pro-inflammatory signaling. In addition, PAR‑2 can be activated by tissue factor (TF)–activated factor V (FVa) complex [19].

Thus, the clinical impact of FXa and thrombin inhibition with non-Vitamin K antagonist oral anticoagulants (NOACs) might go beyond their anticoagulatory effects and involve pleiotropic effects mediated via PAR signaling pathways. Over the past years, such potentially beneficial effects have been described for all NOACs [8, 16, 26, 32]; however, a comprehensive summary of the findings with edoxaban was not yet available. Therefore, this review focuses on potential pleiotropic effects of the FXa-inhibitor edoxaban.

## Potential pleiotropic effects of edoxaban in inflammation, atherosclerosis, and stroke

**Fig. 3:**
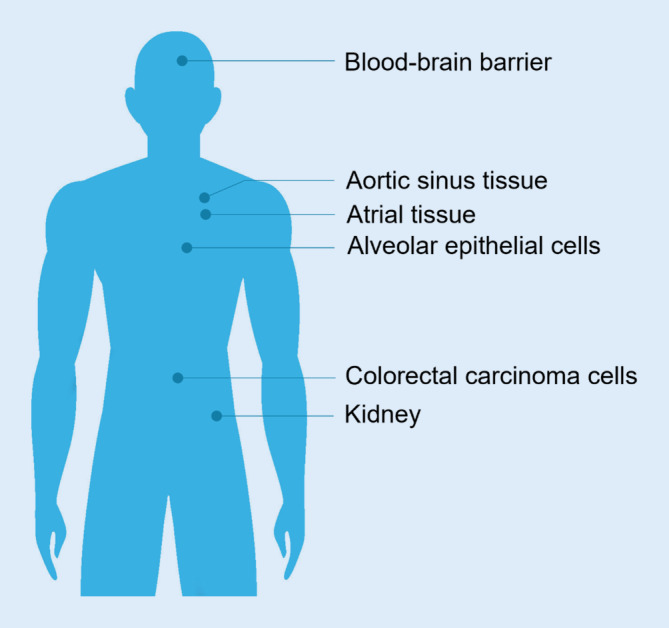
Potential targets for pleiotropic effects of edoxaban in vivo*. *Translational view from the in vitro and animal experiments described in this review to the human organ systems which might potentially be affected by the pleiotropic effects of factor Xa inhibition

Recently, pleiotropic effects of FXa inhibition via edoxaban have been experimentally tested in various in vitro human cell culture models as well as in vivo in different murine and canine models (Fig. 3).

Table 1 summarises the key findings from selected studies investigating the effects of edoxaban on PAR signaling pathways and inflammation.

**Table 1:** Key findings: selected studies investigating the effects of edoxaban on PAR signaling pathways and inflammation

	Test system	Type and number of individuals	Duration	Edoxaban application	Intervention	Main results
Millenaar et al. 2020 [24]	ApoE^−/−^ knockout mice	Ctr: Control (*N* = 3–5)	8 Weeks	Oral/chow	Cholesterol-rich diet and femoral artery ligation	Difference in hind-limb perfusion restoration: no significant difference
		Warf: Warfarin 3 g/kg + vitamin K1 1 g/kg (*N* = 8)				Lowest amounts of plaque tissue and fibrosis in aortic sinus with Edo (plaque tissue: *p* = 0.024 vs. Ctr; *p* = 0.14 vs. Warf; fibrosis: *p* = 0.027 vs. Ctr; *p* = 0.081 vs. Warf)
		Edo: edoxaban 375 mg/kg (*N* = 5–6)				
Almengló et al. 2020 [1]	HUVEC	Ctr: Control (*N* = n.a.)	n.a.	Added to cell culture medium	HUVEC cells ± Edo, FXa, or Edo + FXa	Edo enhanced HUVEC cell viability and growth
		Edo: edoxaban (*N* = n.a.)				Edo counteracted FXa-induced pro-migratory and pro-inflammatory effects
		FXa: factor Xa (*N* = n.a.)				Edo inhibited adhesion of platelets and PBMCs to endothelial cells and counteracted FXa-induced expression of adhesion molecules
		Edo + FXa (*N* = n.a.)				–
Tsujino et al. 2019 [38]	Beagles implanted with ventricular pacemaker	Sham (*N* = 6)	19 Days Edo +14 days ventricular tachypacing	Oral/chow	Ventricular tachypacing (VTP) ± Edo	Prolongation of AF by VTP was suppressed by Edo (*p* < 0.01 vs. Ctr; *p* = n. s. vs. sham)
		Ctr: placebo (*N* = 6)				Increase in atrial fibrotic area by VTP was suppressed by Edo (*p* < 0.01 vs. Ctr)
		Edo: edoxaban 2.0 mg/kg/day (*N* = 6)				Increase in PAR‑2 and in fibronectin expression by VTP was suppressed by Edo (both *p* < 0.05 vs. Ctr)
Mollenhauer and Rudolph (unpublished data)	C57/B6J mice implanted with AngII osmotic minipumps	Ctr (*N* = 5–8)	14 Days	Oral/chow	AngII minipumps (1.5 ng/g/min) ± oral Edo	Edo significantly reduced number and length of AngII-induced AF episodes (*p* < 0.05)
		AngII: angiotensin II (*N* = 5–9)				Edo did not reduce AngII induced fibrosis or enhanced collagen I expression
		AngII + Edo: angiotensin II + edoxaban 0.25 mg/mouse/day (*N* = 5–15)				No changes in PAR‑2 expression by AngII + edoxaban
						Edo significantly attenuated AngII induced increase in expression of thrombopoetin, IL-17, IL‑4, INFγ, IL‑5 and MCP5
Wiedmann et al. 2020 [41]	Human potassium channels expressed in *Xenopus laevis* oocytes	Edoxaban 1 µM (*N* = 7)	n.a.	Added to bath solution	Voltage clamp stimulation ± NOAC	NOACs did not significantly alter peak current amplitudes of potassium channels K_V_11.1, K_V_1.5, K_V_4.3, K_ir_2.1, K_ir_2.2, or K_2P_2.1
		Apixaban 1 µM (*N* = 7)				
		Rivaroxaban 1 µM (*N* = 9)				No significant effect of NOACs on current-voltage relationships
		Dabigatran 1 µM (*N* = 5)				
Bukowska et al. 2020 [5]	Cell lines A549 (human lung carcinoma cells; model for type II alveolar epithelial cells) and Calu3 (human bronchial adenocarcinoma cells)	Ctr: control	n.a.	Added to cell culture medium	A549 or Calu3 cells ± FXa ± Edo, vorapaxar, or GB83	Edo prevented FXa-induced increase in ERK1/2 activation and in the expression of pro-inflammatory cytokines; PAR‑1 inhibitor vorapaxar showed similar effects
		FXa: factor Xa 25 nmol/l				
		FXa + Edo: FXa + edoxaban 1 µmol/l				Edo prevented FXa-induced mitochondrial alteration with restricted capacity for ATP generation; similar effects seen with vorapaxar and GB83 (PAR‑2 inhibitor)
		FXa + Vorapaxar 10 µmol/l				
		FXa + GB83 10 µmol/l				FXa did not increase apoptosis or cause metabolic cellular dysfunction
Bieber et al. 2020 [3]	C57/B6J mice subjected to tMCAO	Ctr: vehicle (*N* = 3–9)	24 h (or 7 days for MRI)	Oral gavage via gastric tube	Induction of stroke by tMCAO and treatment with Edo or VKA	Edo significantly reduced infarct volumes on day 1 after stroke (*p* < 0.001 vs Ctr; *p* < 0.05 vs. VKA)
		Edo: edoxaban 3.3 mg/kg body weight (*N* = 3–9)				Edo improved neurological outcome (*p* < 0.05 vs. Ctr; *p* = n. s. vs. VKA) and function of the blood-brain barrier on day 1 after stroke (*p* < 0.05 vs. Ctr and vs. VKA for leakage into ipsilateral hemisphere)
		VKA: phenprocoumon 0.3 mg/kg body weight (*N* = 3–9)				Edo reduced cerebral expression of pro-inflammatory cytokines IL-1β (*p* < 0.05 vs. Ctr and vs. VKA) and IL‑6 (*p* < 0.01 vs. Ctr, *p* = n. s. vs. VKA)
Oe et al. 2016 [25]	Diabetic nephropathy mouse model (Akita mutation in *insulin2* gene and eNOS knockout)	eNOS^+/+^DM: untreated non-DN control (*N* ≥ 5)	3 Months	Oral	Induction of diabetic nephropathy by *eNOS* knockout and *insulin2* Akita mutation ± Edo	DM increased renal FXa mRNA; knockout of eNOS in DM mice led to diabetic nephropathy with increased mRNA expression levels for pro-inflammatory and pro-fibrotic cytokines *Tgfb, Pai1, Col1, Col4, *and* Tnfa* (*p* < 0.05 vs. eNOS^+/+^DM Ctr for all), and for *Par1* and *Par2* (*p* < 0.05 for *Par*1, *p* = n. s. for *Par*2 vs. eNOS^+/+^DM Ctr)
		eNOS^+/+^DM + Edo: edoxaban-treated non-DN mice (*N* ≥ 5)				Edo significantly reduces *Pai1, Col1, Col4*, and *Tnfa* levels and *Par1* and *Par2* levels in eNOS^−/−^DM mice (*p* < 0.05 vs. eNOS^−/−^DM Ctr for all)
		eNOS^−/−^DM: untreated DN control (*N* ≥ 5)				Edo significantly reduced mesangial matrix score in eNOS^−/−^DM mice (*p* < 0.001 vs eNOS^−/−^DM Ctr)
		eNOS^−/−^DM + Edo: edoxaban (50 mg/kg/day) treated DN mice (*N* ≥ 5)				*Par2* knockout ameliorated DN (shown with additional knockout mouse model)
Hiramoto et al. 2023 [21]	Colon26–inoculated BALB/C mice	Untr: untreated mice	7 Days inoculation period + 21 days treatment	Oral/feeding needle	Inoculation of mice with Colon26 cancer cells ± treatment with NOAC	Edo significantly reduced tumor growth in Colon26-inoculated mice (*p* < 0.01 vs. Ctr); similar effects were seen with Dabi and Riva (*p* < 0.05 vs. Ctr for both)
		Ctr: Colon26-inoculated mice + water				
		Edo: Colon26-inoculated mice + edoxaban 10 mg/kg/mouse (*N* = 5)				TF, PAI‑1, IL‑6, and MMP‑2 plasma levels are significantly elevated in Colon26-inoculated mice (*p* < 0.01 vs. untr for all); Edo significantly reduced these plasma levels in Colon26-inoculated mice (*p* < 0.01 vs. Ctr for all)
		Riva: Colon26-inoculated mice + rivaroxaban 5 mg/kg/mouse (*N* = 5)				PAR‑1 and PAR‑2 expression was significantly increased in Colon26-inoculated mice (*p* < 0.01 vs. untr); Edo significantly reduced PAR‑2, but not PAR‑1 expression levels (*p* < 0.01 vs. Ctr for PAR-1)
		Dabi: Colon26-inoculated mice + dabigatran 50 mg/kg/mouse (*N* = 5)				Edo treatment led to increased apoptosis and elevated p53 protein levels in tumor tissues of Colon26-inoculated mice (*p* < 0.01 vs. Ctr)

*AF* atrial fibrillation, *AngII* angiotensin II, *ApoE* apolipoprotein E, *ATP* adenosine triphosphate, *Col* collagen, *Ctr* control, *Dabi* dabigatran, *DM* diabetes mellitus, *DN* diabetic nephropathy, *Edo* edoxaban, *eNOS* endothelial nitric oxide synthase, *ERK* extracellular signal-regulated kinase, *FXa* factor Xa, *HUVEC* human umbilical vein endothelial cells, *IL* interleukin, *INF* interferon, *MCP5* monocyte chemotactic protein 5, *MMP‑2* matrix metalloproteinase‑2, *MRI* magnetic resonance imaging, *n.a.* not applicable, *NOAC* non-Vitamin K antagonist oral anticoagulant, *n.* *s.* not significant, *Pai1* plasminogen activator inhibitor‑1, *PAR* protease activated receptor, *PBMC* blood mononuclear cells, *Riva* rivaroxaban, *TF* tissue factor, *Tnfa* tumor necrosis factor a, *Tgfb* tumor growth factor beta, *tMCAO* transient middle artery occlusion, *untr* untreated, *VKA* vitamin K antagonist, *VTP* ventricular tachypacing

## The role of FXa inhibition with edoxaban in atherosclerosis and vascular remodeling

Vitamin K antagonists can expedite atherosclerosis and plaque formation due to vascular calcification [35]. In contrast, recent studies in an apolipoprotein E‑deficient (ApoE^−/−^) atherosclerotic mouse model suggest that NOACs do not induce valvular and arterial calcification processes [17, 30]. Interestingly, AF was also seen to interfere with occurrence of cardiac ischemia [11, 12]. The effect of edoxaban in comparison to warfarin on atherosclerosis, vascular remodeling and fibrosis was also investigated in a ApoE^−/−^ mouse model [24]. Hypercholesterolaemia and atherosclerosis were induced by feeding with a cholesterol-rich high-fat diet. Animals received either edoxaban- or control chow. Compared to control, mice receiving edoxaban had significantly reduced amounts of plaque tissue in the aortic sinus and significantly reduced fibrosis. The reduction of fibrosis in Apo E^−/−^ deficient mice was higher with edoxaban compared with warfarin. The reduction in plaque formation showed the same trend for edoxaban. However, mRNA levels of pro-inflammatory cytokines (interleukin 6 [IL-6], interleukin 1β [IL-1β], tumor necrosis factor alpha [TNFα], and monocyte chemotactic protein 1 [MCP-1]) did not show pro-inflammatory effects of warfarin or anti-inflammatory effects of edoxaban in the ApoE^−/−^ mouse model [24]. The authors discuss that this atherosclerosis model might not be sufficient to reveal significant effects in vascular inflammation [24]. However, the positive effects of edoxaban on fibrosis and plaque tissue are supported by findings of vasculo-protective effects of other NOACs [17, 22, 29, 30, 34].

Edoxaban also enhanced human umbilical vein endothelial cell (HUVEC) growth in a dose-dependent manner. It did not show any pro-migratory or angiogenic effects on HUVEC cells itself but was able to counteract FXa-mediated migration of HUVEC cells in a wound healing assay and to partially counteract FXa-induced anti-angiogenic activity in a tube formation assay. These results suggest a stabilising effect of edoxaban on human endothelial cells in the presence of FXa. Functional adhesion of platelets or peripheral blood mononuclear cells (PBMC) to a HUVEC monolayer was tested in absence or presence of edoxaban, FXa or their combination, as PBMC adhesion is associated with atherogenesis and vascular inflammation. Edoxaban inhibited FXa- and TNFα-induced adhesion of platelets or PBMCs to a HUVEC monolayer and was able to reduce both PBMC and platelet adhesion in a concentration-dependent manner even under basal conditions. When further exploring the underlying pathway, mRNA analysis showed a FXa-induced upregulation of the expression of three cell adhesion molecules, intercellular cell adhesion molecule 1 (ICAM-1), vascular cell adhesion molecule 1 (VCAM-1), and E‑selectin (SELE). Edoxaban did not alter these expression levels but counteracted FXa-induced upregulation. The upregulation of VCAM‑1 and SELE seemed to be mediated by PAR‑1 and 2 (in case of SELE only by PAR-2), while ICAM‑1 did not seem to be regulated via the two PAR receptors. For all three adhesion molecules an involvement of NF-κB and PI3K activation was shown. The authors conclude that if these findings are confirmed in an in vivo setting, edoxaban might counteract the effects of pro-inflammatory factors released in the vessel and to reduce platelet adhesion and might therefore contribute to a probable cardioprotective benefit [1].

## Effects of FXa inhibition with edoxaban on atrial fibrosis

Previously, FXa has been shown to be associated with the activation of inflammatory signaling in human atrial tissue, leading to the development of atrial fibrotic remodeling [6]. The effect of edoxaban on atrial fibrosis was studied in a canine model for congestive heart failure [38]. AF was induced in beagles implanted with pacemakers. After implantation, animals received either edoxaban, placebo, or no medication for 19 days. In animals in the edoxaban and placebo groups, but not in sham animals, ventricular tachypacing (VTP) was started 7 days after pacemaker implantation and given for 14 days. After 14 days of VTP, animals in the placebo group showed significantly prolonged duration of AF episodes compared to sham beagles, whereas this effect was attenuated by treatment with edoxaban versus placebo. Inducibility of AF was comparable between the three groups. While VTP significantly increased the atrial fibrotic area in both the edoxaban and the placebo group, the fibrotic response was significantly attenuated with edoxaban versus placebo. As myocardial fibrosis has been strongly associated with FXa and PAR‑2 signaling [2], the investigators measured PAR‑2 expression in the tissue of the left atrial wall and found significantly increased levels in placebo-treated animals compared to sham, whereas there was no increase in PAR‑2 expression in the atria of edoxaban-treated animals. Similar results were observed for expression of fibronectin in atrial wall tissue. The authors concluded that edoxaban can inhibit VTP-induced progression of AF and reduce structural remodeling by suppression of PAR‑2 and fibronectin upregulation as well as atrial fibrosis [38].

**Fig. 4:**
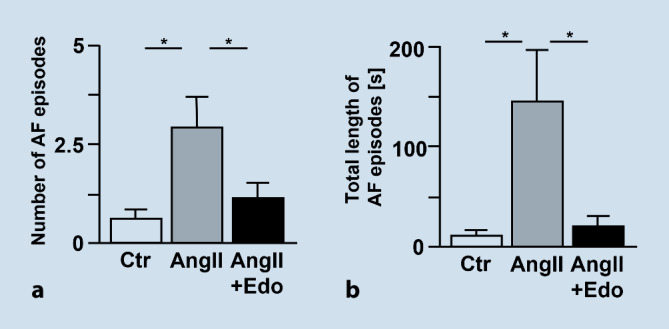
**a** Total number of AF episodes and **b** total length of AF episodes after treatment of C57/B6J wildtype mice with vehicle (0.5% methylcellulose; Ctr, *n* = 8), angiotensin II (1.5 ng/g/min for 2 weeks via osmotic minipump; AngII, *n* = 8), or angiotensin II plus edoxaban (1.5 ng/g/min for 2 weeks via osmotic minipump; edoxaban 0.25 mg/mouse/day in 0.5% methylcellulose orally; AngII + Edo, *n* = 10). An AF episode was defined as a series of repetitive atrial ectopic beats lasting for more than 1 s. *p < 0.05. *AF* atrial fibrillation, *AngII* angiotensin II, *Ctr* control, *Edo* edoxaban

**Fig. 5:**
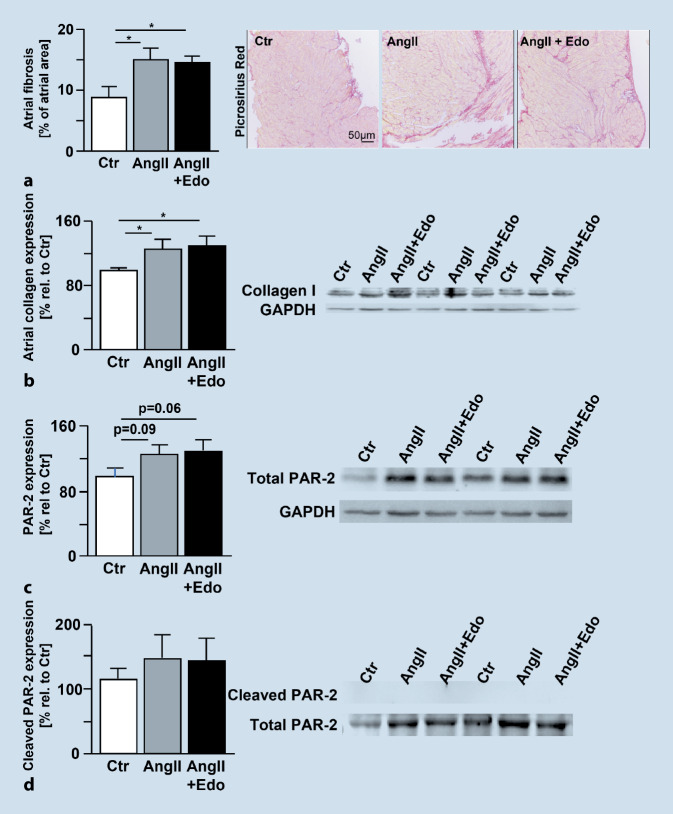
**a** Histochemical evaluation of atrial fibrosis by picrosirius red staining (Ctr, *n* = 6; AngII, *n* = 9; AngII+Edo, *n* = 15). **b** Atrial collagen I expression relative to Ctr as indicated by immunoblotting. (Ctr, *n* = 6; AngII, *n* = 6; AngII + Edo, *n* = 6). *p < 0.05. **c** Total PAR‑2 expression and **d** cleavage (activation) of PAR‑2 as indicated by immunoblotting of control, angiotensin II- or angiotensin II + edoxaban-treated animals (Ctr, *n* = 5; AngII, *n* = 5; AngII + Edo, *n* = 5). No significant differences were detected. C57/B6J wildtype mice treated with vehicle (0.5% methylcellulose; Ctr), angiotensin II (1.5 ng/g/min for 2 weeks via osmotic minipump; AngII), or angiotensin II plus edoxaban (1.5 ng/g/min for 2 weeks via osmotic minipump; edoxaban 0.25 mg/mouse/day in 0.5% methylcellulose orally; AngII + Edo). *AngII* angiotensin II, *Ctr* control, *Edo* edoxaban

**Fig. 6:**
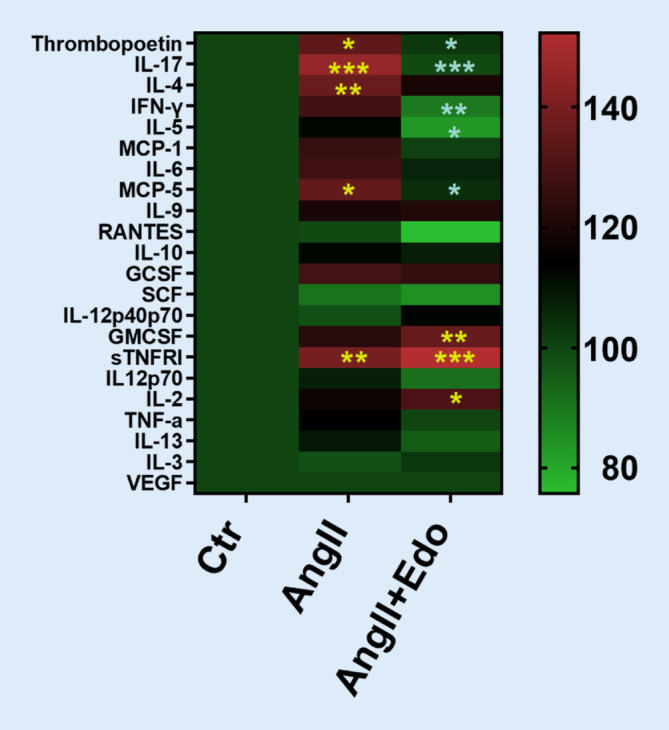
Dot blot array intensity analysis of pooled plasma from Ctr, AngII or AngII + Edo- treated mice (*n* = 6 for all groups), normalised to cytokine levels of Ctr (100%, left panel). *Yellow asterisks*: AngII + Edo vs. Ctr; *grey asterisks*: AngII + Edo vs. AngII; *p < 0.05, **p < 0.01, **p < 0.001. *AngII* angiotensin II, *Ctr* control, *Edo* edoxaban

A murine model of angiotensin II (AngII) -induced AF was used to investigate whether inhibition of FXa with edoxaban has anti-arrhythmic and anti-fibrotic effects and can therefore oppose the development or progression of AF (Mollenhauer and Rudolph, first published here). These data have not been published elsewhere before; therefore, results are described in more detail below. AF was induced in mice by subcutaneous AngII administration via osmotic minipumps. After implantation of the minipumps, mice received cereals impregnated with edoxaban 0.25 mg/animal/day in 0.5% methylcellulose or vehicle solution (0.5% methylcellulose). At 14 days after AngII, electrophysiological atrial stimulation resulted in an increased number and length of AF episodes compared to vehicle-treated animals (Fig. 4a,b). Treatment with edoxaban attenuated AngII-induced vulnerability for AF episodes and reduced duration of AF episodes (*p* < 0.05 for Edo + AngII vs. AngII). In this study, edoxaban did not reduce AngII-induced atrial fibrosis or attenuate AngII-induced atrial collagen I expression (Fig. 5a,b). Immunoblotting of isolated atria showed a trend towards enhanced total and activated PAR‑2 expression upon treatment with AngII, which could not be attenuated by treatment with edoxaban (Fig. 5c,d). However, AngII-mediated increases in plasma cytokine levels of thrombopoetin, interleukin (IL)-17, IL‑4, interferon (INF) γ, IL-5- and MCP‑5 were significantly attenuated with edoxaban (*p* < 0.05 for AngII + Edo vs. AngII) (Fig. 6). These findings suggest an important role of anti-inflammatory processes contributing to the positive effects of edoxaban in AF development. Yet, the underlying mechanism remains unclear.

## Effects of NOACs on atrial repolarizing potassium channels

Treatment with edoxaban has been associated with a significant reduction in death from cardiovascular causes in patients with AF, supported by a tendency to also reduce all-cause death in these patients [10]. In experiments investigating direct effects of edoxaban on potassium channels as potential targets for antiarrhythmic therapy, human genes encoding for cardiac potassium channels K_V_11.1, K_V_1.5, K_V_4.3, K_ir_2.1, K_ir_2.2 and K_2P_2.1 were expressed in *Xenopus laevis* oocytes [41]. Currents were measured in the absence and presence of NOACs using voltage clamp electrophysiology. Edoxaban did not significantly affect peak current amplitudes nor current-voltage relationships of the investigated channels. Similar results were seen for the other NOACs. While direct interactions between NOACs and cardiac potassium channels are not likely to contribute to the positive clinical outcomes, indirect effects on cardiac potassium channels and their potential signaling pathways still need to be investigated further [41].

## FXa as a mediator of pro-inflammatory effects and mitochondrial alterations via PAR signaling in human alveolar cells

Potential anti-inflammatory effects of edoxaban in the development of chronic lung diseases were studied using a human type II alveolar epithelial cell model (A549). The investigators could show that FXa activated extracellular signal-regulated kinase (ERK1/2) and induced increased expression of the pro-inflammatory cytokines IL‑8, ICAM‑1, and MCP‑1 [5]. Vorapaxar, a PAR‑1 inhibitor, and edoxaban were both effective in preventing the induction of FXa-mediated activation of ERK1/2 as well as of IL‑8, ICAM‑1, and MCP‑1. Of note, the PAR‑2 inhibitor GB83 did not counteract FXa-mediated ERK1/2-activation and even had an additional stimulatory effect on the expression of the three inflammatory molecules. As the inflammatory gene expression partly depends on mitochondrial function, the effect of exogenous FXa on mitochondrial oxygen consumption in epithelial cells was measured. FXa reduced mitochondrial oxygen consumption, which is an indicator for restricted capability of ATP generation in mitochondria. Again, administration of edoxaban or vorapaxar prevented the decrease in oxygen consumption and therefore the impairment of mitochondrial function. However, further analysis of caspase3 activity and mRNA expression of apoptosis markers showed that treatment with FXa did not induce apoptosis in two different human lung epithelial cell lines (A549 and Calu3) and did not affect metabolic cellular activity in human epithelial cells [5]. Interestingly, FXa induced intracellular signaling resulting in apoptosis in epithelial tumor cells. These differential findings might be due to a dose-dependency of the cellular effects of FXa [5].

## Anti-FXa-mediated effects on ischaemic stroke severity and blood–brain barrier inflammation

Patients with AF who already experienced a previous ischaemic stroke are at increased risk of cerebrovascular events [31]. Edoxaban has proven to be as effective as warfarin in secondary prevention of ischaemic stroke in patients with atrial fibrillation, while significantly reducing the risk of developing intracranial haemorrhage by approximately 50% compared to warfarin [31]. Anti-inflammatory effects of edoxaban were also seen post-stroke in a murine model [3]. FXa and activation of thrombin could mediate inflammatory processes via PAR‑1, 2 and 4 [27]. PAR‑1 and 4 mediated activation of macrophages and induced pro-inflammatory cytokine expression and dysfunction of the blood–brain barrier [23]. Potential effects of FXa inhibition by edoxaban on inflammation and blood–brain barrier function in acute stroke were studied in the model of transient middle cerebral artery occlusion in mice treated with edoxaban, the vitamin K antagonist phenprocoumon, or vehicle only [3]. Edoxaban significantly reduced the infarct volume compared to phenprocoumon or vehicle. This was accompanied by better neurological and functional outcomes after stroke compared to control. Furthermore, edoxaban significantly reduced blood–brain barrier leakage and ipsilateral formation of brain edema. The cerebral expression of the pro-inflammatory cytokines IL‑6 and IL-1β was significantly reduced with edoxaban. In addition, only treatment with edoxaban but not vitamin K antagonists resulted in a significant reduction in macrophages and microglia invading the brain parenchyma through the blood–brain barrier. Experimental data from this study suggest that treatment with edoxaban has anti-inflammatory and blood–brain barrier protecting effects and might be beneficial in attenuating ischaemic stroke pathology [3].

## The role of factor Xa and PAR2 in diabetic nephropathy

The role of FXa and PAR‑2 in diabetic nephropathy has been investigated in murine and podocyte models. [25]. To model diabetic nephropathy, mice with Akita mutation in the insulin 2 gene and endothelial nitric oxide synthase (eNOS) knockout were used. Diabetes mellitus (DM) increased renal expression of FX mRNA and FXa activity in the murine urine as well as an increase in FXa expression in glomerular macrophages [25]. Inhibition of FXa by administration of edoxaban ameliorated diabetic nephropathy in the eNOS^−/−^ DM mouse model and reduced the expression of the profibrotic and pro-inflammatory genes *Pai1, Col1, Col4, *and* Tnfa* as well as expression of *Par1* and *Par2*. *Par2* expression in the kidneys was increased in eNOS^−/−^ DM mice, and lack of *Par2* was shown to also ameliorate diabetic nephropathy. Interestingly, inhibition of FXa with edoxaban and lack of *Par2* expression did not have additive anti-inflammatory effects. The results suggest that activation of FXa and PAR‑2 worsens diabetic nephropathy through an enhanced inflammatory response. Edoxaban reduced the severity of diabetic nephropathy, but could not fully reverse it [25].

## Effects of FXa inhibition with edoxaban on tumor growth

The suppression of the PAR‑2 signaling pathway via FXa inhibition was subject to a study [21] investigating the effect of NOACs on tumor growth in a colorectal cancer mouse model. Colorectal carcinoma cells (Colon26 cell line) were transplanted into mice by subcutaneous inoculation. Colon26-inoculated mice treated with orally administered edoxaban or other direct oral anticoagulants developed significantly smaller tumors compared to water-treated controls. With edoxaban, tumor volume was reduced to approximately 28% of the tumor volume in control animals. Of note, the tumor size observed in edoxaban-treated animals was also significantly lower than in animals treated with two other NOACs. Further investigations on the potential underlying pathway found that edoxaban significantly decreased plasma levels of the pro-inflammatory factors IL‑6 and matrix metalloproteinase‑2 (MMP-2) in Colon26-inoculated mice compared to water-treated controls, of the pro-tumorigenic factors TF and plasminogen activator inhibitor‑1 (PAI-1), several markers for tumor growth, and PAR‑1 levels (however, not PAR‑2 levels). While apoptosis was increased in the tumor tissue of water-treated Colon26-inoculated mice, edoxaban increased the number of apoptotic cells, as well as the expression level of p53 protein, a transcription factor regulating cell proliferation and apoptosis. The authors hypothesize that inhibition of the PAR‑2 pathway with edoxaban results in an upregulation of p53 and therefore in increased apoptosis in tumor tissue. The authors point out that there have been contradictory findings on the effect of NOACs on tumor growth, so there may be differences between different types of tumors, types of cancer models, and NOACs used [21].

## Perspective

To the authorsʼ knowledge, this is the first in-depth review of pleiotropic effects of edoxaban investigated in vitro and in experimental animal models. Several well-characterised models of pathophysiological settings were applied to explore edoxaban’s effects beyond its anticoagulatory properties.

Throughout the different models and cell types, edoxaban attenuated FXa- and thrombin induced pro-inflammatory and pro-fibrotic effects. Key findings were the reduction of FXa and thrombin-induced increases in pro-inflammatory cytokine expression ([1, 3, 21, 25]; Mollenhauer and Rudolph, first published here). Reductions of PAR‑1 and PAR‑2 levels were found by some, but not all studies. This might be due to different experimental models, cell types and setups used, but also to possible differences in the underlying mechanisms of edoxaban–PAR interaction in different cell types and tissues. It is still unknown to what extent atrial fibrosis is induced by PAR‑2 compared to other potential pathways; however, the findings suggest that edoxaban is a promising target for inhibition of the PAR‑2 pathway [38].

Similar non-haemostatic pleiotropic effects have been described for rivaroxaban, apixaban, and dabigatran previously, confirming the anti-inflammatory and anti-proliferative effects seen in the experiments included in this review [8, 16, 26, 32]. The findings with edoxaban are adding further evidence to the hypothesis that inhibition of FXa and thrombin with NOACs may be beneficial in preventing the progression of atherosclerosis, fibrosis, and other inflammatory conditions.

The test systems used are of broad variety and included in vitro (human epithelial cell culture, human alveolar cell lines, *Xenopus Laevis* oocytes) and animal in vivo experiments (knockout mouse systems, pharmacologically or electrically induced mouse and canine systems, cancer cell-inoculated mouse system). For animal models, also the method of edoxaban application varied between studies (gavage, oral via impregnated food) and one study used vitamin K1 as food supplement for the vitamin K antagonist control group to keep mice healthy. This might also explain differences between the trials regarding the ability of edoxaban to attenuate FXa-induced regulation of cytokines and inflammatory processes. The concentrations of edoxaban used in the different models vary between the studies and differ from the dosages used in AF treatment. PAR receptors vary between different tissues as well as species [19]. Therefore, it is unclear to which extent results seen in in vitro, mouse or canine models can be translated into a therapeutic human setting.

The results of these experimental studies may also translate to clinical findings seen with edoxaban in randomized clinical trials and real-world data on the treatment and prevention of stroke and systemic embolic events: the significant reduction in cardiovascular death [10] and recurrent ischaemic stroke [31] compared to warfarin, or low rates of renal worsening seen with edoxaban after 2 years of treatment [18]. NOACs have also been shown to reduce the risk of stroke and systemic embolic embolism irrespective of the absence or presence of coronary artery disease [42]. Additional in vivo and clinical data will be needed to shed further light into the pleiotropic effects of edoxaban and other NOACs and their potential targets.

## Declarations

Animal studies in mice with results first presented in this review have been performed by M. Mollenhauer and V. Rudolph. All other animal studies included in this review and performed by any of the authors have been published elsewhere previously. All applicable international, national, and institutional guidelines for the care and use of animals were followed. No studies with human participants were performed for this article. All studies mentioned were in accordance with the ethical standards indicated in each case.
